# Design and Fabrication of an Epoxy/Glass Microbeads-Based 1-3 Piezoelectric Composite

**DOI:** 10.3390/mi16040361

**Published:** 2025-03-21

**Authors:** Qiyun Liu, Jinjie Zhou, Ziliang Jia, Pengfei Zhou

**Affiliations:** 1 School of Mechanical Engineering, North University of China, Taiyuan 030051, China; lqynuchina@163.com (Q.L.); 15513722929@163.com (Z.J.); 13073577026@163.com (P.Z.); 2 Shanxi Key Laboratory of Intelligent Equipment Technology in Harsh Environment, Taiyuan 030051, China

**Keywords:** epoxy/glass microbeads-based, ceramic volume fraction, acoustic impedance of the polymer, high electromechanical coupling factor, modified 1-3 piezoelectric composite

## Abstract

An epoxy/glass microbeads-based 1-3 piezoelectric composite is proposed, to enhance electromechanical conversion efficiency. Firstly, based on the series-parallel theory, the theoretical model is established. Secondly, the epoxy resin/glass microbeads-based 1-3 piezoelectric composite is simulated by finite element software. The effects of polymers with different acoustic impedances, the thicknesses of piezoelectric composites, and ceramic volume fractions are analyzed systematically. After parameter optimization, the epoxy/glass microbeads-based 1-3 piezoelectric composite is prepared. The experimental results agree well with the theoretical and simulation results. When the ceramic volume fraction is 60.0%, its electromechanical coupling factor is the largest, which is 0.714. Compared with the prepared traditional 1-3 piezoelectric composites with the same parameters, its electromechanical coupling factor is increased by 7.8%. Therefore, the epoxy/glass microbeads-based 1-3 piezoelectric composite can enhance the sensitivity and resolution of the transducers, which has potential advantages for improving the performance of transducers.

## 1. Introduction

With the increasing demand for piezoelectric ultrasonic transducers, the low electromechanical conversion efficiency and the high acoustic impedance of the PZT ceramics make it difficult to match the acoustic impedance with air, water, and other coupling agents [1,2,3,4]. In addition, their vibration in the thickness direction is easily affected by lateral vibration, which affects the energy conversion efficiency of the piezoelectric ultrasonic transducers. In 1978, Newnham et al. [5] conducted a systematic study on composite materials and proposed the concept of connectivity of the piezoelectric composites. The piezoelectric composites can be divided into 0-0, 0-1, 0-2, 0-3, 1-1, 1-2, 1-3, 2-2, 2-3, and 3-3 types through different connectivity modes between the piezoelectric ceramics and polymer. In the 1-3 piezoelectric composites, the high viscosity and low acoustic impedance of the polymer can effectively inhibit the lateral vibration of the piezoelectric ceramics and improve the electromechanical coupling factor in the thickness direction [6,7] and make them easier to match the acoustic impedances of the coupling agents [8,9]. Therefore, the 1-3 piezoelectric composites have attracted the attention of researchers, and have been widely used in medical, underwater, and non-destructive testing fields [10,11,12,13].

Based on the series-parallel model proposed by Newnham, Chan et al. [14] proposed a theoretical model to predict the electromechanical properties of the single-layer epoxy-based 1-3 piezoelectric composites, which was in good agreement with the experimental results. Guinovart-Díaz et al. [15,16,17] optimized the effective constant of the 1-3 piezoelectric composites based on unidirectional columnar fibers by using an asymptotic homogenization method and listed specific analytical expressions. Li et al. [18] designed a 1-3-2 piezoelectric composite, the theoretical results showed that its electromechanical coupling factor was slightly lower than that of the traditional 1-3 piezoelectric composite. In addition, some researchers modified the composition of the piezoelectric ceramic. Ma et al. [19] used Li, Ta, and Sb modified (Na, K)NbO_3_ compositions as lead-free piezoelectric ceramic, and prepared the 1-3 piezoelectric composite by using a method of fiber arrangement and epoxy cast, the experimental results showed that the electromechanical coupling factors were in the range of 0.55~0.60. Shen et al. [20] developed a fine-grained Pb-free (Na_0.535_K_0.485_)_0.95_Li_0.05_(Nb_0.8_Ta_0.2_)O_3_ (NKLNT) piezoelectric ceramic by spark plasma sintering (SPS) technique and prepared the NKLNT/epoxy 1-3 piezoelectric composite. The experimental results showed that its electromechanical coupling factor was 0.65. As the polymer of the single-layer 1-3 piezoelectric composite, the density and acoustic impedance of the epoxy resin are still large so its electromechanical properties are less improved.

In order to design and manufacture 1-3 piezoelectric composites with more excellent properties, researchers added silicone rubber with low density and low impedance as a polymer and designed the structures with multiple layers cascade [21,22,23,24,25]. Zhong et al. [26] designed a 1-3 piezoelectric composite with double-layer polymers using PZT-5 ceramic; experimental results showed that when the volume fraction of silicone rubber was at its maximum, its electromechanical coupling factor was 0.67. Zhang et al. [27] designed a 1-3 piezoelectric composite material with a sandwich polymer using PZT-5H ceramic and established a theoretical model. The experimental results showed that when the volume fraction of silicone rubber exceeded 50.0%, the electromechanical coupling factor was 9.8% higher than that of the 1-3 piezoelectric composite. On the basis of the 1-3 piezoelectric composite material with a sandwich polymer, Zhang et al. [28] replaced the epoxy resin with PZT-5A ceramics to increase the stability of the structure and retained the silicone rubber layer in the sandwich polymers and designed a three-layer 1-3-type piezoelectric composite, which has an electromechanical coupling factor of 0.71. However, the above complex structures may put forward higher requirements for machining accuracy.

Researchers are increasingly looking at alternative material systems with lower impedance properties [29,30,31]. He et al. [32] developed an air-based 1-3 piezoelectric composite using 3D printing technology. The simulation results showed that the electromechanical coupling factor was 0.70 when the air volume fraction was 30.0% and the ceramic volume fraction was 40.0%. Jing et al. [33] used PZT-5H ceramics and designed a 3-2 ceramic–air composite by replacing silicone rubber with air and studied the effect of thickness on its electromechanical properties. The experimental results showed that its electromechanical coupling factor was 0.71. In addition, due to the characteristics of low density [34], low dielectric constant [35], and high strength [36], the hollow glass microbeads have excellent acoustic properties and attracted the attention of researchers [37,38,39,40]. Lu et al. [41] studied the mechanism of the impact of the hollow glass microbeads on the properties of the epoxy resin composites. Kang et al. [42] added hollow glass microbeads to epoxy resin and developed an air-coupled acoustic matching layer that had a characteristic acoustic impedance of 1.08 MRayl. Zhou et al. [43] proposed a model to predict the acoustic properties of the epoxy/glass microbeads composite. Liu et al. [44] studied the effect of the mass ratio of hollow glass microbeads on the electromechanical properties of 1-3 piezoelectric composite and successfully prepared 1-3 piezoelectric composites based on epoxy/glass microbeads composites. The test results showed that its electromechanical coupling factor increased to 0.64.

The epoxy/glass microbead composite as a polymer can not only support the piezoelectric column but also reduce the transverse coupling of the piezoelectric ceramic columns. Therefore, we use it as a polymer for a single-layer 1-3 piezoelectric composite. However, the mechanism of the influence of acoustic impedance of polymer composites on piezoelectric properties is still unclear. More importantly, geometric and material parameters have not been investigated for the piezoelectric properties of piezoelectric composites in the epoxy/glass microbeads composite system. Therefore, it is necessary to study the influence of these parameters to reveal the internal relationship of piezoelectric composites and improve the performance of piezoelectric composites. To solve the above problems, the theoretical model and simulation model of epoxy/glass microbeads-based 1-3 piezoelectric composites are established by using series-parallel theory and finite element software. The effects of polymers with different acoustic impedances, overall thicknesses and ceramic volume fractions on the electromechanical properties of the piezoelectric composite are systematically studied. After parameter optimization, the epoxy/glass microbeads-based 1-3 piezoelectric composites were prepared and tested.

## 2. Models of the Epoxy/Glass Microbeads-Based 1-3 Piezoelectric Composite

### 2.1. Theoretical Model

**Figure 1:**
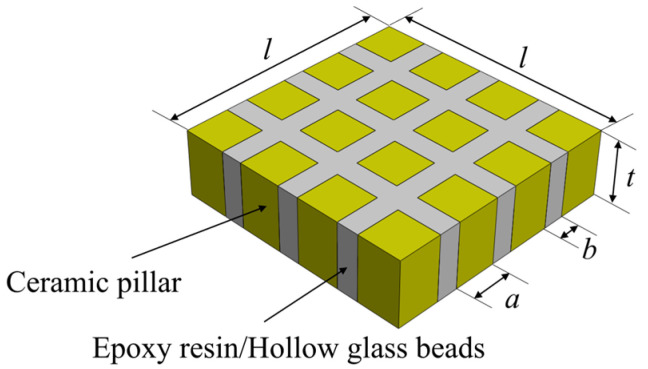
The structure of the epoxy/glass microbeads-based 1-3 piezoelectric composite.

The epoxy/glass microbeads-based 1-3 piezoelectric composites are composed of piezoelectric ceramic and an epoxy/glass microbead mixture. In order to make it vibrate along the thickness direction, exciting voltages are applied to the upper and lower surfaces of the piezoelectric composite. Compared with the PZT-4 ceramics and PZT-5H ceramics, the PZT-5A ceramics have a high piezoelectric factor and good thermal stability. For the filling materials of piezoelectric composites, epoxy resin 618 has become the mainstream choice for general-purpose epoxy resins due to its low viscosity, high strength, and easy processing. Therefore, in this study, the PZT-5A ceramics are selected as piezoelectric materials and epoxy resin 618 is selected as one of the main materials of the polymer. The hollow glass microbeads BR20 with low density and low acoustic impedance are added to epoxy resin 618. The mechanical properties of the glass microbeads used in this study are shown in Table 1. Because hollow glass beads have a very low acoustic impedance, the polymer with low density and low impedance values can be obtained by mixing with epoxy resin, which can effectively suppress the effect of lateral vibration on the vibration of piezoelectric ceramic pillars in the thickness direction. Epoxy/glass microbead mixtures with different acoustic impedances can be obtained by adjusting the ratio of each component. In order to study the effect of the acoustic impedance of the epoxy/glass microbead mixture on the electromechanical properties of the epoxy/glass microbead-based 1-3 piezoelectric composites, three kinds of epoxy/glass microbead mixtures with different acoustic impedances were prepared as polymers, as shown in Table 2. The structure of the epoxy/glass microbeads-based 1-3 piezoelectric composites is shown in Figure 1, and the specific material parameters of the PZT-5A ceramic, the epoxy/glass microbeads mixtures, and the epoxy resin are shown in Table 3. l represents the width of the piezoelectric composite and t represents its thickness. a represents the width of the PZT-5A ceramic pillar and b represents the width of the polymer. For the epoxy/glass microbeads-based 1-3 piezoelectric composite, the ceramic volume fraction $v_{c}$ can be expressed as $a^{2}/{\left(a+b\right)}^{2}$. In order to better study the electromechanical properties of the epoxy/glass microbeads-based 1-3 piezoelectric composite, the relevant theoretical analysis is carried out.

According to generalized Hooke’s law, the elastic constitutive relation in an elastic body is $\left[T\right]=\left[c\right]\left[S\right]$. Because of the piezoelectric effect, piezoelectric ceramics will also be deformed under the action of an external electric field. The deformation will change with the change in the direction and intensity of the electric field; the piezoelectric equation is

$$\left\{\begin{array}{c} \left[T\right]=\left[c^{E}\right]\left[S\right]-{\left[e\right]}^{Τ}\left[E\right]\\ \left[D\right]=\left[e\right]\left[S\right]+\left[\varepsilon^{S}\right]\left[E\right] \end{array}\right.$$

The crystal system of the PZT-5A ceramic is hexagonal. If the polymer is regarded as an isotropic material, its crystal system is cubic. So according to the above piezoelectric equation, their piezoelectric constitutive relations are Equations (1) and (2), respectively

(1) $$\left[\begin{array}{c} T_{1}^{c}\\ T_{2}^{c}\\ T_{3}^{c}\\ T_{4}^{c}\\ T_{5}^{c}\\ T_{6}^{c}\\ D_{1}^{c}\\ D_{2}^{c}\\ D_{3}^{c} \end{array}\right]=\left[\begin{array}{ccccccccc} c_{11}^{E} & c_{12}^{E} & c_{13}^{E} & 0 & 0 & 0 & 0 & 0 & -e_{31}\\ c_{12}^{E} & c_{11}^{E} & c_{13}^{E} & 0 & 0 & 0 & 0 & 0 & -e_{31}\\ c_{13}^{E} & c_{13}^{E} & c_{33}^{E} & 0 & 0 & 0 & 0 & 0 & -e_{33}\\ 0 & 0 & 0 & c_{44}^{E} & 0 & 0 & 0 & -e_{15} & 0\\ 0 & 0 & 0 & 0 & c_{44}^{E} & 0 & -e_{15} & 0 & 0\\ 0 & 0 & 0 & 0 & 0 & \frac{1}{2}\left(c_{11}^{E}-c_{12}^{E}\right) & 0 & 0 & 0\\ 0 & 0 & 0 & 0 & e_{15} & 0 & \varepsilon_{11}^{S} & 0 & 0\\ 0 & 0 & 0 & e_{15} & 0 & 0 & 0 & \varepsilon_{11}^{S} & 0\\ e_{31} & e_{31} & e_{33} & 0 & 0 & 0 & 0 & 0 & \varepsilon_{33}^{S} \end{array}\right]\left[\begin{array}{c} S_{1}^{c}\\ S_{2}^{c}\\ S_{3}^{c}\\ S_{4}^{c}\\ S_{5}^{c}\\ S_{6}^{c}\\ E_{1}^{c}\\ E_{2}^{c}\\ E_{3}^{c} \end{array}\right]$$

(2) $$\left[\begin{array}{c} T_{1}^{m}\\ T_{2}^{m}\\ T_{3}^{m}\\ T_{4}^{m}\\ T_{5}^{m}\\ T_{6}^{m}\\ D_{1}^{m}\\ D_{2}^{m}\\ D_{3}^{m} \end{array}\right]=\left[\begin{array}{ccccccccc} c_{11}^{m} & c_{12}^{m} & c_{12}^{m} & 0 & 0 & 0 & 0 & 0 & 0\\ c_{12}^{m} & c_{11}^{m} & c_{12}^{m} & 0 & 0 & 0 & 0 & 0 & 0\\ c_{12}^{m} & c_{12}^{m} & c_{11}^{m} & 0 & 0 & 0 & 0 & 0 & 0\\ 0 & 0 & 0 & c_{44}^{m} & 0 & 0 & 0 & 0 & 0\\ 0 & 0 & 0 & 0 & c_{44}^{m} & 0 & 0 & 0 & 0\\ 0 & 0 & 0 & 0 & 0 & c_{44}^{m} & 0 & 0 & 0\\ 0 & 0 & 0 & 0 & 0 & 0 & \varepsilon_{11}^{m} & 0 & 0\\ 0 & 0 & 0 & 0 & 0 & 0 & 0 & \varepsilon_{11}^{m} & 0\\ 0 & 0 & 0 & 0 & 0 & 0 & 0 & 0 & \varepsilon_{11}^{m} \end{array}\right]\left[\begin{array}{c} S_{1}^{m}\\ S_{2}^{m}\\ S_{3}^{m}\\ S_{4}^{m}\\ S_{5}^{m}\\ S_{6}^{m}\\ E_{1}^{m}\\ E_{2}^{m}\\ E_{3}^{m} \end{array}\right]$$

where T represents the stress tensor, S represents the strain tensor, D represents the electrical displacement, E represents the electric field, c represents the elastic constant, e represents the piezoelectric constant, and $\varepsilon^{S}$ represents the dielectric constant. Superscripts c and m represent the PZT-5A ceramic and polymer, respectively.

Considering that the epoxy/glass microbeads-based 1-3 piezoelectric composite vibrates along the thickness direction, the thickness direction is defined as the z direction. According to Smith’s assumptions [45], the simplified constitutive relation of the epoxy/glass microbeads-based 1-3 piezoelectric composite is obtained, as shown in Equation (3)

(3) $$\left[\begin{array}{c} {\bar{T}}_{1}\\ {\bar{T}}_{3}\\ {\bar{D}}_{3} \end{array}\right]=\left[\begin{array}{cc} {\bar{c}}_{13}^{E} & -{\bar{e}}_{31}\\ {\bar{c}}_{33}^{E} & -{\bar{e}}_{33}\\ {\bar{e}}_{33} & {\bar{\varepsilon }}_{33}^{S} \end{array}\right]\left[\begin{array}{c} {\bar{S}}_{3}\\ {\bar{E}}_{3} \end{array}\right]$$

where

(4) $${\bar{c}}_{13}^{E}=\frac{v_{c}c_{13}^{E}\left(c_{11}^{m}+c_{12}^{m}\right)+\left(1-v_{c}\right)c_{12}^{m}\left(c_{11}^{E}+c_{12}^{E}\right)}{v_{c}\left(c_{11}^{m}+c_{12}^{m}\right)+\left(1-v_{c}\right)\left(c_{11}^{E}+c_{12}^{E}\right)}$$

(5) $${\bar{e}}_{31}=\frac{v_{c}e_{31}\left(c_{11}^{m}+c_{12}^{m}\right)}{v_{c}\left(c_{11}^{m}+c_{12}^{m}\right)+\left(1-v_{c}\right)\left(c_{11}^{E}+c_{12}^{E}\right)}$$

(6) $${\bar{c}}_{33}^{E}=-\frac{2v_{c}\left(1-v_{c}\right){\left(c_{13}^{E}-c_{12}^{m}\right)}^{2}}{v_{c}\left(c_{11}^{m}+c_{12}^{m}\right)+\left(1-v_{c}\right)\left(c_{11}^{E}+c_{12}^{E}\right)}+v_{c}c_{33}^{E}+\left(1-v_{c}\right)c_{11}^{m}$$

(7) $${\bar{e}}_{33}=-\frac{2v_{c}\left(1-v_{c}\right)e_{31}\left(c_{13}^{E}-c_{12}^{m}\right)}{v_{c}\left(c_{11}^{m}+c_{12}^{m}\right)+\left(1-v_{c}\right)\left(c_{11}^{E}+c_{12}^{E}\right)}+v_{c}e_{33}$$

(8) $${\bar{\varepsilon }}_{33}^{S}=\frac{2v_{c}\left(1-v_{c}\right){\left(e_{31}\right)}^{2}}{v_{c}\left(c_{11}^{m}+c_{12}^{m}\right)+\left(1-v_{c}\right)\left(c_{11}^{E}+c_{12}^{E}\right)}+v_{c}\varepsilon_{33}^{S}+\left(1-v_{c}\right)\varepsilon_{11}^{m}$$

In order to facilitate the analysis of the vibration of the epoxy/glass microbeads-based 1-3 piezoelectric composite along the thickness direction, Equation (3) is replaced by Equation (9)

(9) $$\left[\begin{array}{c} {\bar{T}}_{3}\\ {\bar{E}}_{3} \end{array}\right]=\left[\begin{array}{cc} {\bar{c}}_{33}^{D} & -{\bar{h}}_{33}\\ -{\bar{h}}_{33} & {\bar{\beta }}_{33} \end{array}\right]\left[\begin{array}{c} {\bar{S}}_{3}\\ {\bar{D}}_{3} \end{array}\right]$$

where

(10) $${\bar{c}}_{33}^{D}={\bar{c}}_{33}^{E}+\frac{{\left({\bar{e}}_{33}\right)}^{2}}{{\bar{\varepsilon }}_{33}^{S}}$$

(11) $${\bar{h}}_{33}=\frac{{\bar{e}}_{33}}{{\bar{\varepsilon }}_{33}^{S}}$$

(12) $${\bar{\beta }}_{33}=\frac{1}{{\bar{\varepsilon }}_{33}^{S}}$$

When the epoxy/glass microbeads-based 1-3 piezoelectric composite works along the thickness direction, its resonant frequency $f_{s}$ and anti-resonant frequency $f_{p}$ can be expressed as Equations (13) and (14), respectively. In addition, its effective density ρ is the weighted average of the PZT-5A ceramic and polymer, as shown in Equation (15)

(13) $$f_{s}=\frac{1}{2t}\sqrt{\frac{{\bar{c}}_{33}^{D}}{\rho }\left(1-\frac{8{\left({\bar{h}}_{33}\right)}^{2}}{\pi^{2}{\bar{c}}_{33}^{D}{\bar{\beta }}_{33}^{S}}\right)}$$

(14) $$f_{p}=\frac{1}{2t}\sqrt{\frac{{\bar{c}}_{33}^{D}}{\rho }}$$

(15) $$\rho =v_{c}\rho_{c}+\left(1-v_{c}\right)\rho_{m}$$

where ${\bar{c}}_{33}^{D}$ is the elastic constant component of the epoxy/glass microbeads-based 1-3 piezoelectric composite under constant electrical displacement, ${\bar{h}}_{33}$ is its piezoelectric stiffness constant, ${\bar{\beta }}_{33}^{S}$ is its dielectric impedance component under constant strain, ${\bar{c}}^{E}$ is its elastic stiffness tensor under constant strain and a constant electric field, $\rho_{c}$ is the density of the PZT-5A ceramic, and $\rho_{m}$ is the density of the polymer.

The electromechanical coupling factor reflects the strength of the energy conversion in the process of piezoelectric effect and inverse piezoelectric effect. Since the working modes of the 1-3 piezoelectric composites are mostly vibration along the thickness direction, the electromechanical coupling factor $k_{t}$ in the thickness direction is one of the important indexes to evaluate the overall performance of the epoxy/glass microbeads-based 1-3 piezoelectric composite, and $k_{t}$ can be expressed as Equation (16).

(16) $$k_{t}=\frac{{\bar{h}}_{33}}{\sqrt{{\bar{c}}_{33}^{D}{\bar{\beta }}_{33}}}$$

Due to the large difference between the acoustic impedances of the piezoelectric material and coupling agents, the excited ultrasonic waves will be attenuated sharply in the coupling agents. The piezoelectric material with lower impedance can be more easily matched with the coupling agents and reduce the energy attenuation. Therefore, the acoustic impedance z of piezoelectric material is also an important evaluation index to measure its performance. In the epoxy/glass microbeads-based 1-3 piezoelectric composite, the acoustic velocity c is defined as Equation (17). According to Equations (17) and (18), the acoustic impedance z can be expressed as Equation (19).

(17) $$c=\sqrt{\frac{{\bar{c}}_{33}^{D}}{\rho }}$$

(18) z=ρc

(19) $$z=\sqrt{\rho {\bar{c}}_{33}^{D}}$$

### 2.2. Finite Element Model

**Figure 2:**
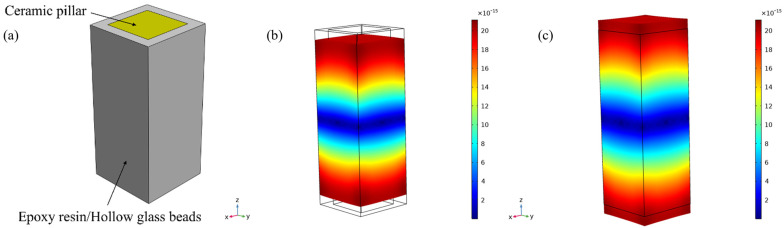
Epoxy/Hollow glass microbeads-based 1-3 piezoelectric composite. (**a**) Finite element model (**b**) Compressed state (**c**) Elongation state.

**Figure 3:**
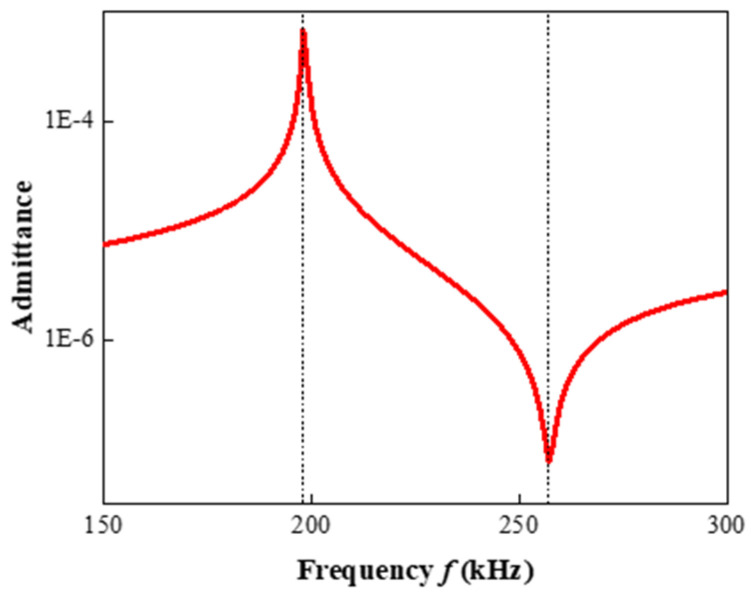
Admittance curve of the epoxy/glass microbeads-based 1-3 piezoelectric composite.

The epoxy/glass microbeads-based 1-3 piezoelectric composites were simulated by the finite element method. Since the arrangement of the PZT-5A ceramic pillars was periodic, in order to simplify the calculation, only one element model of the composite was established in this study, as shown in Figure 2a, and the element width was fixed at 2.3 mm. A voltage of 1 V was applied to the upper surface of this element model, and a voltage of 0 V was applied to its lower surface. The very thin conductive layer was ignored, and then frequency domain analysis was performed. The piezoelectric effect was selected as the multiphysics field. The mesh at the ceramic–polymer interface was encrypted and symmetric constraints were applied to both sides of the model to simulate the mechanical vibration mode of the piezoelectric composite more accurately. Firstly, the characteristic frequency analysis was carried out to observe the vibration modes of each order, as shown in Figure 2b,c. Due to the vibration of the PZT-5A ceramic pillar, the epoxy/glass microbead polymer also vibrates along with it. Meanwhile, the vibration displacements of the upper and lower layers are larger than that of the middle layer. Then, the frequency domain analysis was carried out; Figure 3 shows the admittance curve of the epoxy/glass microbeads-based 1-3 piezoelectric composite obtained by simulation. The peak and valley of the admittance curve represent the resonant frequency $f_{s}$ and anti-resonant frequency $f_{p}$, respectively. According to the IEEE piezoelectric standards [46], the electromechanical coupling factor $k_{t}$ is calculated by Equation (20). According to Equations (14) and (17), the acoustic velocity c can be expressed as Equation (21). The acoustic impedance z is calculated by Equation (18).

(20) $$k_{t}=\sqrt{\frac{\pi f_{s}}{2f_{p}}\cdot \tan\frac{\pi }{2}\left(1-\frac{f_{s}}{f_{p}}\right)}$$

(21) $$c=2f_{p}t$$

## 3. Preparation of the Epoxy/Glass Microbeads-Based 1-3 Piezoelectric Composite

**Figure 4:**
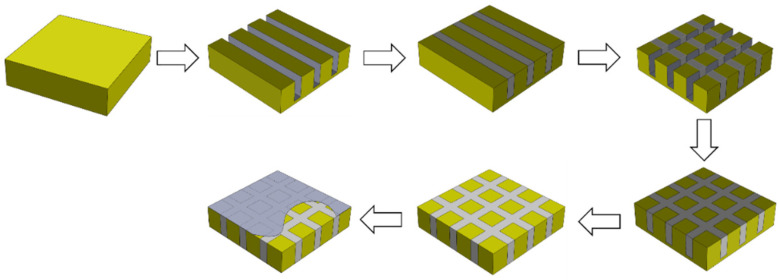
Preparation process of the epoxy/glass microbeads-based 1-3 piezoelectric composite.

Based on the above theoretical and simulation analyses, the feasibility of the epoxy/glass microbeads-based 1-3 piezoelectric composite was verified. The epoxy/glass microbeads-based 1-3 piezoelectric composites were prepared by modified cut-and-fill method, as shown in Figure 4. The cutting machine was SYJ-400 CNC (Shenyang Kejing Auto-instrument Co., Ltd., Shenyang, China). Firstly, the PZT-5A ceramic was cut along the X-axis. The mixture of epoxy resin 618 (Shanghai Aotun Chemical Technology Co., Ltd., Shanghai, China), hollow glass beads BR20(Dongguan Hengyuan New Materials Co., Ltd., Dongguan, China), and Curing agents 593 was prepared in a specific proportion. An appropriate amount of diluent 501 was added to prevent bubbles during the curing process. After mixing the mixture thoroughly, it is evenly filled into the cutting groove. To further remove the bubbles, the preparation is placed in a vacuum incubator for vacuuming and constant temperature curing. After the mixture had been cured, the above steps were repeated along the Y-axis. This modified cut-and-fill method can prevent the PZT-5A ceramic pillars from breaking during the cutting process. Finally, after the second curing the epoxy/glass microbeads-based 1-3 piezoelectric composite was ground to the desired thickness, and its upper and lower surfaces were coated with conductive layers. In addition, although the bottom of the groove filled with epoxy/glass microbeads mixture formed a cavity, this problem was solved by grinding away the bottom of the groove.

**Figure 5:**
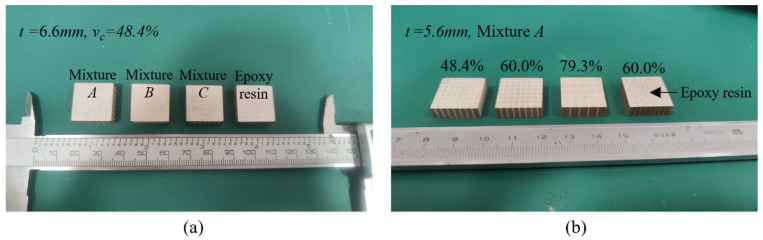
The prepared epoxy/glass microbeads-based 1-3 piezoelectric composites. (**a**) Piezoelectric composites with different polymers (**b**) Piezoelectric composites with different ceramic volume fractions.

Both the length and width of the prepared epoxy/glass microbeads-based 1-3 piezoelectric composites are 18.0 mm, as shown in Figure 5. Figure 5a shows the prepared epoxy/glass microbeads-based 1-3 piezoelectric composites with different polymers, all with thicknesses of 6.6 mm and ceramic volume fractions of 48.4%. The epoxy/glass microbeads-based 1-3 piezoelectric composites with different ceramic volume fractions are shown in Figure 5b. Their polymers are mixture *A*, and their thickness is 5.6mm. In Figure 5b, the first piece on the right is a prepared traditional 1-3 piezoelectric composite with a volume fraction of 60.0% and a thickness of 5.6mm. The resonant frequencies and anti-resonant frequencies of the epoxy/glass microbeads-based 1-3 piezoelectric composites were measured by the UC710 impedance analyzer (Changzhou Tonghui Electronics Co., Ltd., Changzhou, China), as shown in Table 4, Table 5 and Table 6. In the process of preparation, the shape of grooves will appear wide at the top and narrow at the bottom [47] and there is a processing error. In addition, there are some errors in impedance measurements and thickness measurements of the piezoelectric composites. Therefore, there is a slight deviation between the experimental results and theoretical and simulation results. The acoustic impedance z and electromechanical coupling factor $k_{t}$ can be calculated by Equations (18) and (20), respectively.

## 4. Effect Factors of the Epoxy/Glass Microbeads-Based 1-3 Piezoelectric Composite

### 4.1. Effect of the Polymers with Different Acoustic Impedance

When the acoustic impedance of the polymer is changed, its elastic constant and density will be changed, which has a direct effect on the electromechanical properties of the 1-3 piezoelectric composite.

**Figure 6:**
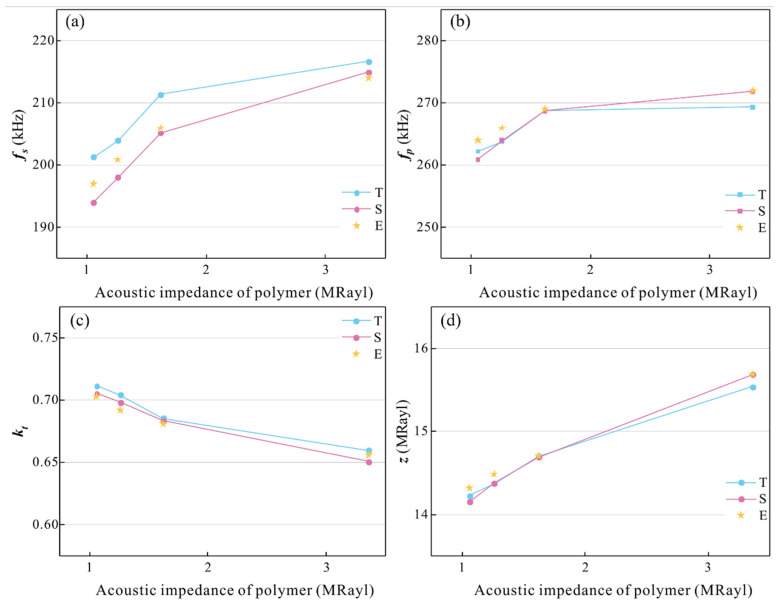
Changing curves of the electromechanical properties with different polymers. (**a**) Resonant frequency $f_{s}$. (**b**) Anti-resonant frequency $f_{p}$. (**c**) Electromechanical coupling factor $k_{t}$. (**d**) Acoustic impedance z.

In order to reveal its physical mechanism and further improve the properties of the piezoelectric composite, the relationships between them were studied by theory, simulation, and experiment. In the epoxy/glass microbeads-based 1-3 piezoelectric composites, the ceramic volume fraction was fixed at 48.4%, the thickness was fixed at 6.6mm, and the polymer was selected as mixtures *A*, *B*, and *C*. In addition, epoxy resin was also used as the polymer for comparison. The results are shown in Table 4, and the changing curves of the electromechanical properties are shown in Figure 6. Because the model is idealized and does not consider the actual processing factors, the theoretical results cannot be completely consistent with the experimental results.

Figure 6a,b show the changing curves of the resonant frequency and anti-resonant frequency. T, S, and E represent theoretical, simulation, and experimental results, respectively. The resonant frequency and anti-resonant frequency increase with the increase of the acoustic impedance of the polymer. This is because when the acoustic impedance of the polymer increases, its elastic constants $c_{11}^{m}$ and $c_{12}^{m}$ increase, so that the elastic constant component ${\bar{c}}_{33}^{D}$ and dielectric impedance component under constant strain ${\bar{\beta }}_{33}^{S}$ increases, and the piezoelectric stiffness constant ${\bar{h}}_{33}$ decreases. The ${\bar{c}}_{33}^{D}$ is much higher than ρ, so according to Equation (13), the resonant frequency is also increased. According to Equation (16), with the increase in the acoustic impedance of the polymer, the electromechanical coupling factor decreases, as shown in Figure 6c. Figure 6d shows the changing curve of the acoustic impedance of the piezoelectric composite. As the acoustic impedance of the polymer increases, its density increases. According to Equation (15), the density of the piezoelectric composite also increases. Therefore, according to Equation (19), the acoustic impedance of the piezoelectric composite increases with the increase in the acoustic impedance of the polymer.

As is shown in Figure 6, the experimental results are in good agreement with the theoretical and simulation results. This shows that a polymer with low acoustic impedance can effectively improve the electromechanical conversion efficiency of the 1-3 piezoelectric composite and reduce its acoustic impedance. The electromechanical coupling factor of the 1-3 piezoelectric composite filled with mixture *A* is 0.702 and the acoustic impedance is only 14.3 MRayl. Therefore, in the next research, mixture A will be used as its polymer.

### 4.2. Effect of Different Thicknesses

**Figure 7:**
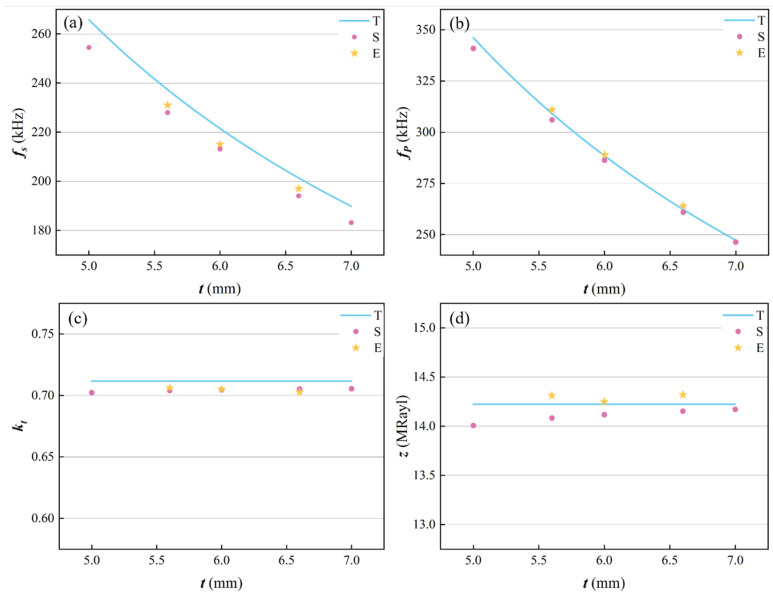
Changing curves of the electromechanical properties with different thicknesses. (**a**) Resonant frequency $f_{s}$. (**b**) Anti-resonant frequency $f_{p}$. (**c**) Electromechanical coupling factor $k_{t}$. (**d**) Acoustic impedance z.

In order to study the effect of the epoxy/glass microbeads-based 1-3 piezoelectric composites with different thicknesses, its thickness was selected as 5.0 mm, 5.6 mm, 6.0 mm, 6.6 mm, and 7.0 mm for the simulation model, and the ceramic volume fraction was still 48.4%. In order to verify the theoretical and simulation results, experimental thicknesses of 5.6 mm, 6.0 mm, and 6.6 mm were selected. Their results are shown in Table 5 and changing curves of the electromechanical properties are shown in Figure 7.

As shown in Figure 7a,b, the resonant frequency and anti-resonant frequency of the epoxy/glass microbeads-based 1-3 piezoelectric composite decrease with increasing thickness. The reason is that the ${\bar{c}}_{33}^{D}$, ${\bar{\beta }}_{33}^{S}$, ${\bar{h}}_{33}$, and ρ are not related to the thickness of the piezoelectric composite. According to Equations (13) and (14), its resonant frequency and anti-resonant frequency are negatively correlated with the thickness of the epoxy/glass microbeads-based 1-3 piezoelectric composite. Figure 7c,d show the changing curves of the electromechanical coupling factor and acoustic impedance. According to Equations (16) and (19), they are not related to the thickness of the piezoelectric composite. Therefore, the electric coupling factor and acoustic impedance remain unchanged with the thickness of the piezoelectric composite. The simulation results of the acoustic impedance of the epoxy/glass microbeads-based 1-3 piezoelectric composite slightly increase with the increase in thickness, as shown in Figure 7d. This is because the ratio between the width and height of the element model in the simulation can slightly affect its simulation results. As the width of the piezoelectric composite is fixed at 2.3 mm, this ratio decreases with the increase in the thickness of the piezoelectric composite, so that the element model becomes elongated gradually. When the element model is more elongated, the vibration in the thickness direction can be obtained more purely. Therefore, the simulation results of acoustic impedance increase slightly and gradually approach the theoretical value.

The experimental results verify the rule of the theoretical results. The resonant frequency and the anti-resonant frequency decrease with the increase in thickness, and the electromechanical coupling factor and acoustic impedance do not change with thickness. It provides a basis for the future design of high electromechanical conversion efficiency and specific frequency of the epoxy/glass microbeads-based 1-3 piezoelectric composite.

### 4.3. Effect of Different Ceramic Volume Fractions

**Figure 8:**
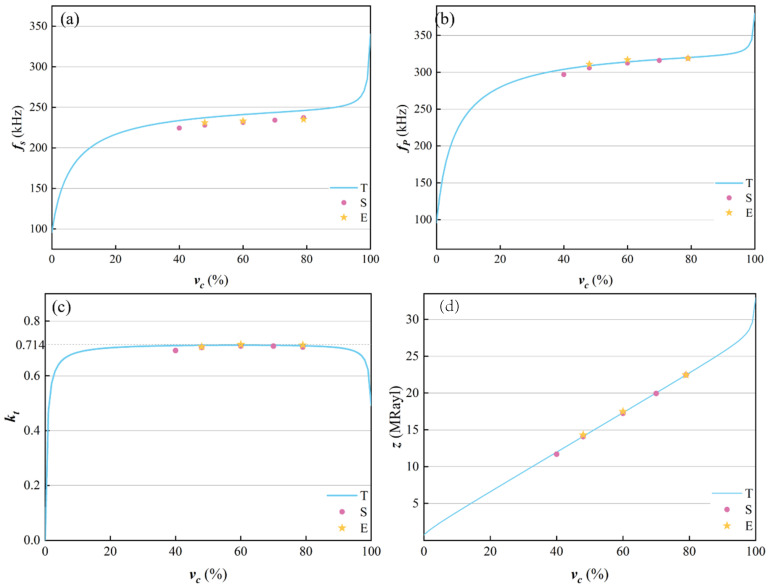
Changing curves of the electromechanical properties with different ceramic volume fractions. (**a**) Resonant frequency $f_{s}$. (**b**) Anti-resonant frequency $f_{p}$. (**c**) Electromechanical coupling factor $k_{t}$. (**d**) Acoustic impedance z.

According to the analysis in Section 4.2, the thickness does not change the electromechanical coupling factor and acoustic impedance of the epoxy/glass microbeads-based 1-3 piezoelectric composite, so its thickness was fixed at 5.6mm. The ceramic volume fraction is also one of the important factors affecting the electromechanical properties of the epoxy/glass microbeads-based 1-3 piezoelectric composite. In order to analyze its effect, the ceramic volume fraction was selected as 40.0%, 48.4%, 60.0%, 70.0%, and 79.3% in the simulation model. The experimental ceramic volume fractions of 48.4%, 60.0%, and 79.3% were selected to verify the theoretical and simulation results. Their results are shown in Table 6 and the changing curves of the electromechanical properties are shown in Figure 8.

Figure 8a,b show the changing curves of resonant frequency and anti-resonant frequency. According to Equation (14), when the thickness is fixed, the anti-resonant frequency is only related to the ${\bar{c}}_{33}^{D}$ and ρ. Although both the ${\bar{c}}_{33}^{D}$ and ρ increase as the ceramic volume fraction increases, the growth rate of the ${\bar{c}}_{33}^{D}$ is bigger than that of the ρ, and the value of the ${\bar{c}}_{33}^{D}$ is much bigger than that of the ρ. Therefore, when the ceramic volume fraction increases, the anti-resonant frequency increases. According to Equation (13), the resonant frequency also increases. The changing curve of the electromechanical coupling factor is shown in Figure 8c. With the increase in ceramic volume fraction, theoretical, simulation, and experimental results firstly increase and then decrease, and reach the maximum when the ceramic volume fraction is 60%. When the ceramic volume fraction increases, the product of the ${\bar{c}}_{33}^{D}$ and ${\bar{\beta }}_{33}^{S}$ firstly decreases and then increases, but the ${\bar{h}}_{33}$ firstly decreases and then increases. According to (16), the electromechanical coupling factor first decreases and then increases. The simulation and experimental results are consistent with theoretical results. Because only piezoelectric ceramics have a piezoelectric effect, the electromechanical coupling factor is low when the ceramic volume fraction is too small. When the ceramic volume fraction is too large, the gaps between the ceramic pillars are narrow. Since the filled polymer cannot support them well play a coupling role, the electromechanical coupling factor is also low. Moreover, with the increase in ceramic volume fraction, it gradually decreases to the electromechanical coupling factor of the PZT-5A ceramic. The acoustic impedance is shown in Figure 8d. According to Equation (19), the acoustic impedance increases with the increase in the ceramic volume fraction. The experimental results are in good agreement with the theoretical and simulation results. In addition, when the ceramic volume fraction ranges from 48.4% to 79.3%, the experimental value of the electromechanical coupling factor can reach above 0.70.

### 4.4. Effect of Different Polymers on Different Volume Fractions

**Figure 9:**
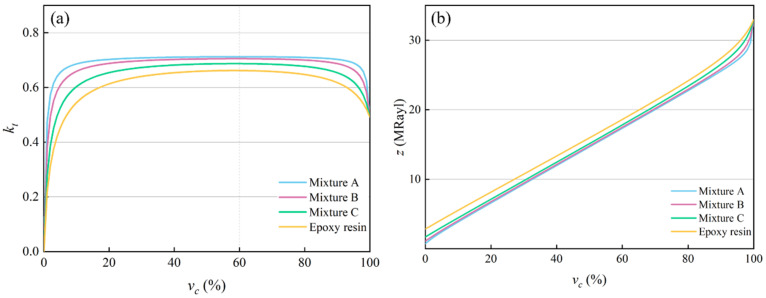
Changing curves of the electromechanical properties. (**a**) Electromechanical coupling factor $k_{t}$. (**b**) Acoustic impedance z.

For piezoelectric composites filled with mixtures *A*, *B*, *C*, and epoxy resin, the effects of different ceramic volume fractions on their electromechanical properties were analyzed theoretically. The theoretical results are shown in Figure 9. Figure 9a,b show the changing curves of the electromechanical coupling factor and acoustic impedance. When different polymers are filled, the electromechanical coupling factors of the piezoelectric composites reach the maximum value when the ceramic volume fraction is 60.0%, as shown in Table 7. In each stage of the ceramic volume fraction, with the decrease in the acoustic impedance of the polymer, the electromechanical coupling factor increases and the acoustic impedance decreases.

**Figure 10:**
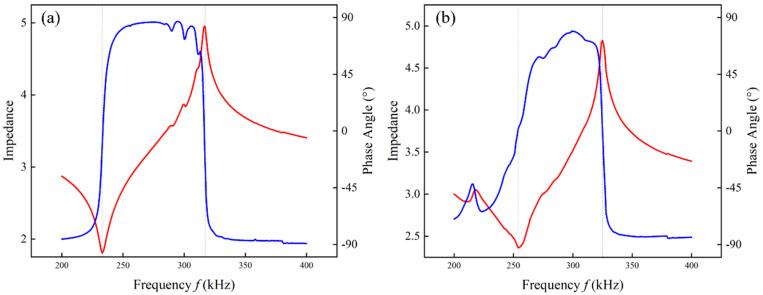
Performance tests of the prepared 1-3 piezoelectric composites. (**a**) Epoxy/glass microbeads-based. (**b**) Traditional epoxy-based.

To verify the above theoretical analysis, A traditional 1-3 piezoelectric composite with a thickness of 5.6mm and a ceramic volume fraction of 60.0% was prepared and compared with an epoxy/glass microbeads-based 1-3 piezoelectric composite with mixture *A*. The experimental results are shown in Figure 10. The epoxy/glass microbeads mixture with low impedance can obtain lower resonant and anti-resonant frequencies. Their electromechanical properties are shown in Table 8. The experimental results of the electromechanical coupling factor and acoustic impedance agree with the theoretical results in Table 7. Compared with the traditional 1-3 piezoelectric composite with the same dimension parameters, the electromechanical coupling factor of the epoxy/glass microbeads-based 1-3 piezoelectric composite is raised to 0.714, which is increased by 7.8%, and the acoustic impedance is reduced by 6.3%. This is because the elastic constant and density of the epoxy/glass microbeads mixture are smaller than that of the epoxy resin, making the ${\bar{c}}_{33}^{D}$ of the epoxy/glass microbeads-based 1-3 piezoelectric composite smaller than that of the traditional 1-3 piezoelectric composite. In addition, the properties of the 1-3 piezoelectric composite in this study were compared with those of other piezoelectric composites. Table 9 shows that the optimization research of multiple parameters is meaningful to promote the improvement of its performance.

## 5. Conclusions

Based on the series-parallel theory, a theoretical model of the epoxy/glass microbeads-based 1-3 piezoelectric composite was established. The effects of the polymers with different acoustic impedance, different thicknesses and ceramic volume fractions were analyzed by theory and simulation. The experimental results verified the accuracy of the theoretical and simulation results. The following conclusions were obtained:

(1) The theoretical, simulation, and experimental results show that with the increase in the acoustic impedance of the polymer, the resonant frequency and anti-resonant frequency of the epoxy/glass microbeads-based 1-3 piezoelectric composite increase, and its acoustic impedance value also increases, but the electromechanical coupling factor decreases. When the thickness of the epoxy/glass microbeads-based 1-3 piezoelectric composites increases, the resonant frequency and anti-resonant frequency decrease and the electromechanical coupling factor and acoustic impedance are almost unchanged.
(2) The resonant frequency and anti-resonant frequency of the epoxy/glass microbeads-based 1-3 piezoelectric composite increase with the increase in the ceramic volume fraction, and the electromechanical coupling factor reaches 0.714 when the ceramic volume fraction is 60.0%.
(3) Compared with the traditional 1-3 piezoelectric composite under the same dimension parameters, the electromechanical coupling factor of the epoxy/glass microbeads-based 1-3 piezoelectric composite is raised to 0.714, which is increased by 7.8%. Therefore, it can achieve higher sensitivity and resolution of the transducers, which has potential advantages for improving the performance of transducers.

## Figures and Tables

**Table 1:** Mechanical properties of the hollow glass microbeads.

Hollow Glass Microbeads	Real Density *ρ* (kg/m^3^)	Wall Thickness (μm)	Acoustic Impedance Z(MRayl)
BR20	200	0.5–1	0.456

**Table 2:** The volume fractions of the glass microbeads in the epoxy resin.

Epoxy/Glass Microbeads Mixture	Volume Fraction (%)
*A*	27.5%
*B*	22.0%
*C*	18.8%

**Table 3:** Material parameters of PZT-5A ceramic and polymers.

Parameters	PZT-5A	Mixture *A*	Mixture *B*	Mixture *C*	Epoxy Resin 618
$\rho_{c}\left({\mathrm{kg}/m}^{3}\right)$	7750	694	727	756	1200
$Z\left(10^{6}\mathrm{Rayl}\right)$	32.93	1.06	1.26	1.62	3.36
$c_{11}\left(10^{10}{N/m}^{2}\right)$	12.1	0.08	0.17	0.39	0.68
$c_{12}\left(10^{10}{N/m}^{2}\right)$	7.54	0.04	0.08	0.2	0.42
$c_{13}\left(10^{10}{N/m}^{2}\right)$	7.52	-	-	-	-
$c_{33}\left(10^{10}{N/m}^{2}\right)$	10.6	-	-	-	-
$e_{31}\left({C/m}^{2}\right)$	−5.4	-	-	-	-

**Table 4:** Theoretical, simulation, and experimental results with different polymers.

Polymer		Mixture *A*	Mixture *B*	Mixture *C*	Epoxy Resin
$f_{s}$ (kHz)	Theory	201.33	204.02	204.02	216.71
	Simulation	194.06	198.04	198.04	214.98
	Experiment	197.00	201.00	206.00	214.00
$f_{p}$ (kHz)	Theory	262.24	263.85	263.85	269.35
	Simulation	260.92	264.00	264.00	271.88
	Experiment	264.00	266.00	269.00	272.00

**Table 5:** Simulation and experimental results with different thicknesses.

t (mm)		5.0	5.6	6.0	6.6	7.0
$f_{s}$ (kHz)	Simulation	254.48	227.96	213.1	194.06	183.15
	Experiment	-	231.00	215.00	197.00	-
$f_{p}$ (kHz)	Simulation	340.88	306.01	286.29	260.92	246.33
	Experiment	-	311.00	289.00	264.00	-

**Table 6:** Simulation and experimental results with different ceramic volume fractions.

$v_{c}$ (%)		40.0	48.4	60.0	70.0	79.3
$f_{s}$ (kHz)	Simulation	224.34	227.96	231.41	234.08	237.3
	Experiment	-	231.00	233.00	-	235.00
$f_{p}$ (kHz)	Simulation	296.94	306.01	312.6	316.1	318.97
	Experiment	-	311.00	317.00	-	319.00

**Table 7:** Theoretical results with different polymers ($v_{c}=60\%$).

Polymer	$k_{t}$	z **(MRayl)**
Mixture *A*	0.712	17.33
Mixture *B*	0.705	17.48
Mixture *C*	0.687	17.81
Epoxy resin	0.662	18.60

**Table 8:** Electromechanical performance comparison.

	*t* (mm)	*v_c_* (%)	*f_s_* (kHz)	*f_p_* (kHz)	*k_t_*	*z* (MRayl)
Epoxy/glass microbeads-based	5.6	60.0	233.00	317.00	0.714	17.49
Traditional epoxy resin-based	5.6	60.0	254.00	325.00	0.662	18.67

**Table 9:** Comparison of the developed 1-3 piezocomposite with other piezocomposites.

Common Structure Types	$k_{t}$
Epoxy/glass microbeads-based 1-3 piezoelectric composite	0.714
Air-based 1-3 piezoelectric composite [32]	0.70
3-2 Ceramic–Air Composite [33]	0.71
1-3 piezoelectric composites with three-layer cascade [28]	0.71

## Data Availability

Data are contained within the article.
